# Sodium Tanshinone II Sulfonate A Ameliorates Hypoxia-Induced Pulmonary Hypertension

**DOI:** 10.3389/fphar.2020.00687

**Published:** 2020-05-21

**Authors:** Ya-Ru Bao, Jing-Wei Chen, Yan Jiang, Lin-Hui Wang, Rong Xue, Jin-Xian Qian, Guo-Xing Zhang

**Affiliations:** ^1^Department of Physiology, Medical College of Soochow University, Suzhou, China; ^2^Department of Internal Medicine, Suzhou TCM Hospital affiliated to Nanjing University of Chinese Medicine, Suzhou, China; ^3^Department of Respiratory and Critical Care Medicine, Affiliated Suzhou Hospital of Nanjing Medical University, Suzhou Municipal Hospital, Suzhou, China

**Keywords:** hypoxic pulmonary hypertension, sodium tanshinone II sulfonate A (STS), apoptosis, PI3K/Akt//mTOR signaling pathway, autophagy, inflammatory factors

## Abstract

**Background:**

Pulmonary hypertension (PH) remains a prevalent disease globally. Sodium tanshinone II sulfonate A (STS) has been used in clinical treatment of PH.

**Aims:**

The aim of the present study was to investigate the effect of sodium STS treatment on hypoxia-induced PH and related mechanisms.

**Methods:**

Male Sprague-Dawley rats were housed in a hypoxic chamber with an oxygen concentration of 10 ± 1% for 8 h a day over 21 days. Rats were treated with either STS (low-dose: 10 mg/kg or high-dose: 30 mg/kg) or LY294002 (which is an inhibitor of PI3K). Pulmonary arterial pressure (PAP) was measured, right ventricular hypertrophy parameters were monitored, lung edema parameters were measured, and pathological changes were observed by hematoxylin-eosin (HE) staining. Protein expressions of apoptosis, and PI3K/AKT/mTOR/autophagy pathways in rat lung tissue were examined by western blot. Levels of the pro-inflammatory factors IL-6, IL-8, TNF-α in lung tissues of rats were measured using an enzyme linked immunosorbent assay (ELISA).

**Results:**

Results of our study demonstrate that persistent exposure to hypoxic conditions increased PAP, right ventricular hypertrophy, lung edema, parameters of lung vascular proliferation and decreased the ratio of Bax/Bcl-2. Furthermore, hypoxic conditions activated the PI3K/Akt/mTOR pathway, inhibited autophagy, and elevated abundance of inflammatory factors in rat lung tissue. Treatment with STS resulted in a dose-dependent decrease in PAP, right ventricular hypertrophy, lung edema, lung vascular proliferation and reversed hypoxia induced lung tissue protein expression and pro-inflammatory factors in rat lung tissue. In addition, hypoxia-induced increases in PAP, cardiac hypertrophy, and lung expression of the proteins PI3K/Akt/mTOR/autophagy pathway were partially reversed by treatment with LY294002.

**Conclusions:**

STS alleviates hypoxia-induced PH by promoting apoptosis, inhibiting PI3K/AKT/mTOR pathway, up-regulating autophagy, and inhibiting inflammatory responses.

## Introduction

Pulmonary hypertension (PH) is a disease of the lung which is characterized by increased pulmonary vascular resistance, vascular remodeling, and obstruction of the pulmonary arteries. PH can lead to right heart failure and death (Humbert et al., 2019). The World Health Organization (WHO) has classified PH into five groups on the basis of similarities in pathophysiology, clinical presentation, and therapeutic options: Group I: pulmonary arterial hypertension, Group II: pulmonary hypertension due to left heart disease, Group III: pulmonary hypertension due to lung disease and/or chronic hypoxia, Group IV: pulmonary hypertension due to blood clots in the lungs, Group V: pulmonary hypertension due to blood and other disorders (Simonneau et al., 2019). PH related secondary effects due to lung disease and/or chronic hypoxia is termed Class III PH and is the second most common cause of elevated pulmonary arterial pressure following heart disease (Strange et al., 2012; Lopez-Campos et al., 2016). Pulmonary vascular remodeling and vasoconstriction are the two main pathological features seen in patients presenting with group III PH. Despite recent advances in treatment of patients with class III PH using pulmonary vasodilators, the clinical outcomes in these patients continue to be disappointing (Young et al., 2019). New therapeutic approaches which target changes in lung parenchyma, pulmonary vasculature remodeling, and vasoconstriction should be explored for their ability to improve clinical outcomes in patients presenting with Group III PH.

The molecular mechanisms of group III PH remain unclear and controversial. Nonetheless, several common pathways of pathogenesis have been suggested over the decades. Initially, increased contraction of the pulmonary artery is observed, which is due to alteration of potassium and calcium channel flux due to changes in the redox status of vascular smooth muscle cells (Dunham-Snary et al., 2017). Following changes in redox balance, reduced bioactivity of nitric oxide (NO) is apparent (Bailey et al., 2010), followed by release of various vasoconstrictors (Kourembanas et al., 1991) and increases in the sensitivity of myofilament calcium (Weigand et al., 2011). Subsequently, hypoxia-inducible factors (HIFs) are activated, which mediate vessel tissue cell proliferation, migration, phenotype transition and further promotes vasoconstriction of vessels by increasing the release of vasoconstrictors and decreasing the release of vasodilators (Young et al., 2019). In addition, activation of HIF triggers inflammation of the lungs leading to lung edema and aggregation of lung tissue, ultimately resulting in hypoxia (Eltzschig and Carmeliet, 2011). To the best of our knowledge several approaches have been implemented to manage symptoms of patients presenting with Group III PH such as use of vasodilators, HIFs, and targeted drug therapy for inhibiting inflammatory signaling pathway and increasing lung ventilation. However, despite the range of treatments used on patients, 3 year survival remains as low as 33% for patients with mean pulmonary artery pressures >40 mmHg (Strange et al., 2012; Hurdman et al., 2013; Gall et al., 2017).

Sodium tanshinone II sulfonate A (STS) is one of the major active constituents of *Salvia miltiorrhiza* Bunge. STS has been widely used in China for clinical treatment of patients presenting with various microcirculatory disturbance-related diseases (Gao et al., 2012; Li et al., 2018). Recent studies have demonstrated that STS can inhibit proliferation of pulmonary smooth muscle cells and stimulates expression of Kv2.1 (Huang et al., 2009). Treatment with STS inhibits expression of canonical transient receptor pulmonary arterial smooth muscle cells derived from a rat model of pulmonary hypertension (Wang et al., 2013a). In addition, STS inhibits hypoxia-induced enhancement of store-operated calcium entry (SOCE) in pulmonary arterial smooth muscle cells *via* activation of the PKG-PPAR-γ signaling axis (Jiang et al., 2016). Moreover, a clinical trial monitoring the efficacy of STS for treatment of PH patients demonstrated its therapeutic effects (Wang et al., 2013b). Although there is evidence demonstrating beneficial effects of STS for treatment of class III PH, detailed mechanisms of its therapeutic effects remain little understood. Therefore, in the current study we used a rat model of hypoxia induced PH and observed pathological and biochemical responses following treatment with STS.

## Materials and Methods

### Animal Model

Ten-week-old male Sprague-Dawley rats were purchased from Shanghai Laboratory Animal Center (Shanghai, China). Rats were housed in optimal conditions with standard hygiene. Temperature was maintained at 25°C with a 12/12 light/dark cycle. Rats were fed standard rat chow and water *ad libitum*. Experiments were performed in accordance with the National Institutes of Health Guidelines for the Use of Laboratory Animals (NIH, publication number 85-23, revised 1996.), and performed according to guidelines for the care and use of animals established by Soochow University.

### Hypoxia-Induced Pulmonary Hypertension Model

Hypoxia was maintained using a XF-2CL unit (Nanjing Xingfei analytical instruments Co. LTD, Nanjing, China). Concentration of O_2_ in the rat chamber was controlled by infusion of N_2_. CO_2_, water vapor, and ammonia were removed *via* air pump to maintain the desired atmosphere of the hypoxia chamber. Rats were randomized into five treatment groups: (1) Normoxia group: In this group rats were placed under normal atmospheric condition (n = 10); (2) Hypoxia group: In this group rats were placed in the hypoxic chamber (oxygen concentration of 10%) for 8 h per day for 3 weeks (n = 10), then rats were returned to normal atmospheric condition (16 h per day), as described previously (Lu et al., 2017); (3) Hypoxia with low dose treatment of STS group (STS was obtained from Shanghai NO.1 Biochemical & Pharmaceutical Co., Ltd, Shanghai, China, batch No: 1606212): In this group, rats were placed in the hypoxic chamber (oxygen concentration 10%) for 8 h per day for 3 weeks and received 10 mg/kg STS per day *via* intraperitoneal injection after exposure to hypoxic conditions for 8 h (n = 10); (4) Hypoxia with high dose treatment of STS group: In this group, rats were placed in the hypoxic chamber (oxygen concentration 10%) for 8 h per day for 3 weeks and administered 30 mg/kg STS per day *via* intraperitoneal injection (n = 10), as previously conducted (Pan et al., 2017); (5) Hypoxia with treatment using LY294002 group (LY294002 was purchased from Cell Signaling Technology Co., LTD, Shanghai, China, Catalog No: 99015): In this group rats were placed in hypoxic chambers (oxygen concentration 10%) for 8 h per day for 3 weeks and then treated using 0.03 mg/kg LY294002 per day *via* intraperitoneal injection (n = 10) (Zhou et al., 2018).

### Measurement of Pulmonary Artery Pressure

After 3 weeks of treatment, rats were anesthetized with 10% chloral hydrate (350 mg/kg i.p.), and pulmonary artery pressure was measured using a heart performance analysis system (ALCBIO, Shanghai Alcott Biotech CO., LTD, Shanghai, China). A polystyrene PE-50 catheter (Bunzl Healthcare CO., LTD, London, UK) was inserted into the pulmonary artery *via* the right common carotid artery, and the other end was connected to the measurement system. Mean pulmonary artery pressure was electronically measured. Rats were then sacrificed and position of the catheter in the pulmonary artery was confirmed. Lung and heart tissue were harvested for further analysis.

### Measurement of Right Ventricle Hypertrophy

Rats were sacrificed and hearts were sampled. The right ventricle was isolated and weighed. The remaining heart tissue, including left ventricle and septum, were weighed. The ratio of right ventricle to left ventricle, and septum weight was calculated.

### Measurement of Lung Edema

Lungs of rats were collected at sacrifice. The left upper lobe of rat lung was washed with phosphate-buffered saline (PBS), dried with filter paper, weighed, and then placed in an oven for 48 h at 60°C. Next, samples were weighed and ratio of dry weight of lung sample to its wet weight was calculated.

### Histological Analysis

The upper right lobe of the lung was fixed and embedded in paraffin. Specimens were cut into 5-µm sections, stained with hematoxylin-eosin (HE), and observed using a digital light microscope (Olympus, Tokyo, Japan). Vascular hypertrophy parameters were assessed *via* the ratio of medial artery wall thickness to medial artery outer diameter, and the ratio of the medial artery wall area to the medial artery area.

### Western Blotting

Lung tissue was homogenized with radio immunoprecipitation assay (RIPA) buffer (50 mm Tris, pH 7.0, 150 mM NaCl, 1% Triton-X-100) containing phenylmenthanesulfonyl fluoride (R&D Systems Inc., Minneapolis, US). Homogenates were centrifuged at 12,000 × g for 10 min at 4°C. Cell protein was separated using SDS-PAGE and transferred to polyvinylidene fluoride (PVDF) membranes (Hybond TM-ECL; Amersham Pharmacia Biotech, Inc. Shanghai, China). Membranes were blocked in 5% nonfat milk in PBS and 0.1% Tween-20 at room temperature. Blots were then incubated with primary antibodies: anti-Bcl-2 antibody (1:1,000, Immunoway Biotech, Inc., Shanghai, China, Catalog No: YM3795), anti-Bax antibody (1:1,000, abcam, Inc., Shanghai, China, Catalog No: ab182734), anti-PI3 antibody (1:1,000, abcam, Inc., Shanghai, China, Catalog No: ab140307), anti-phosphorylated Akt antibody (1:1,000, abcam, Inc., Shanghai, China, Catalog No: ab38449), anti-mTOR antibody (1:1,000, abcam, Inc., Shanghai, China, Catalog No: ab32028), anti-phosphorylated mTOR antibody (1:1,000, abcam, Inc., Shanghai, China, Catalog No: ab137133), anti-phosphorylated S6K1 antibody (1:1,000, abcam, Inc., Shanghai, China, Catalog No: ab47379), anti-S6K1 antibody (1:1,000, abcam, Inc., Shanghai, China, Catalog No: ab32359), anti-Beclin-1 (1:1,000, Santa Cruz Biotech, Inc., Shanghai, China, Catalog No: sc-48341), anti-LC3 (1:1,000, abcam, Inc., Shanghai, China, Catalog No: sc-376404), anti-p62 (1:1,000, abcam, Inc., Shanghai, China, Catalog No: ab56416), or anti-GAPDH (Santa Cruz Biotech, Inc., Shanghai, China, Catalog No: sc-47724). Next, membranes were incubated for 1 h with a secondary antibody (HRP-conjugated anti-rabbit Ig-G, 1:2,000, abcam, Inc., Catalog No: ab205718). Membranes were then washed for 15 min using TBS-T, three times. Washing was done to remove excess antibody before incubation for 1 min with chemiluminescent reagents (ECL, R&D Systems Inc, Minneapolis, USA). Membranes were subsequently exposed to X-ray film. Immunoreactive bands were detected by analysis of X-ray films using the software Image J (Rawak Software Inc., Stuttgart, Germany). The quantity of target proteins was normalized to GAPDH expression.

### Measurement of Inflammatory Factors

Lung tissue was homogenized to measure concentration of IL-6 (Catalog No: E-EL-R0015c), IL-8 (Catalog No: E-EL-H0048c), and TNF-α (Catalog No: E-EL-R0019c) using commercially available enzyme-linked immunosorbent assay (ELISA) kits (Elabscience, Inc., Shanghai, China). All steps were performed according to the manufacturer's instructions provided with the ELISA kit.

### Statistical Analysis

The SPSS 18.0 software (IBM, Inc., Shanghai, China) was used for statistical analysis. Data are presented as mean ± standard error mean (S.E.M). Grouped data were analyzed using one-way analysis of variance followed by the Student-Newman-Keuls test. A *P* value < 0.05 was considered statistically significant.

## Results

### Effect of Treatment with STS on Pulmonary Artery Pressure

**Figure 1:**
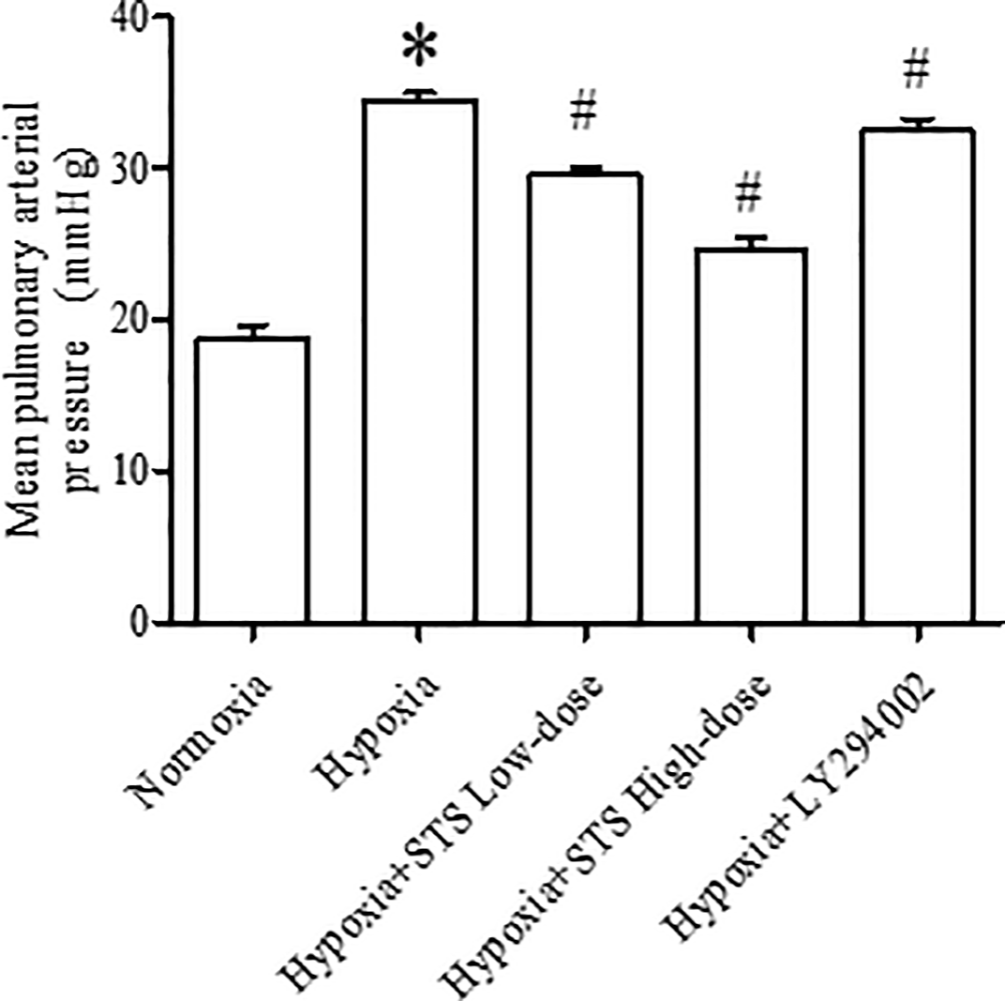
Mean pulmonary artery pressure in rats. Data are presented as mean ± SEM (n = 10). *P < 0.05 compared with normoxia group, ^#^*P* < 0.05, compared with hypoxia group.

When compared with the normoxia group, rats exposed to 3-week hypoxia had significantly greater pulmonary artery pressure (34.50 ± 0.47 *vs.* 18.80 ± 0.85 mmHg, *P* < 0.001). Treatment with STS after hypoxic exposure for the low (10 mg/kg per day) and high dose (30 mg/kg per day) resulted in significant reductions in hypoxia induced increases in pulmonary artery pressure when compared with the hypoxia group (29.52 ± 0.53, 24.65 ± 0.77 *vs.* 34.50 ± 0.47 mmHg, *P* < 0.001). These results confirm that STS effectively reduces hypoxia-induced increases in pulmonary artery pressure. In addition, treatment with LY294002 significantly reduced hypoxic induced increases in pulmonary artery pressure when compared with the hypoxia group (32.44 ± 0.82 *vs.* 34.50 ± 0.47 mmHg, *P* < 0.05) (**Figure 1**). These results suggest that a key role is played in the pathogenesis of hypoxia-induced pulmonary artery hypertension by the PI3/Akt/mTOR signaling pathway.

### Effect of Treatment With STS on Hypoxia-Induced Right Ventricular Hypertrophy

**Figure 2:**
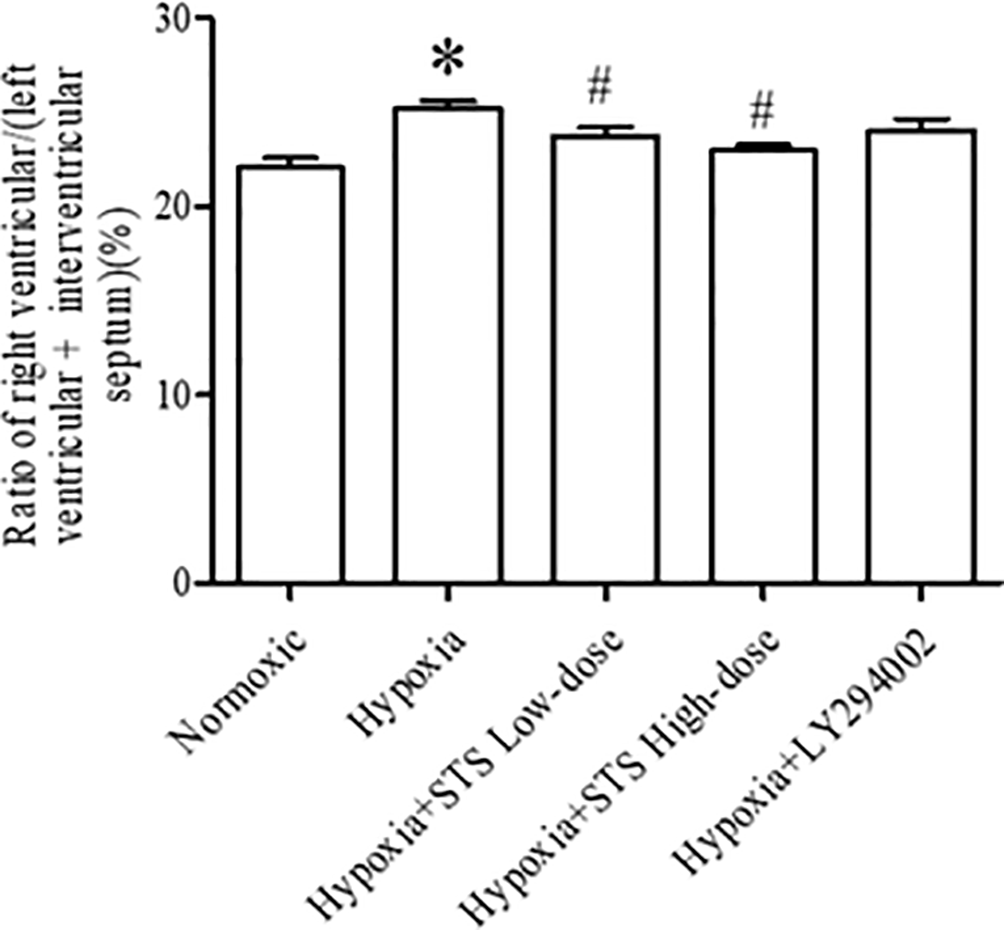
Ratio of right ventricular/(left ventricular + interventricular septum) for each group. Data are presented as mean ± SEM (n = 8). *P < 0.05 compared with normoxia group, ^#^*P* < 0.05, compared with hypoxia group.

Right ventricular hypertrophy was calculated by measuring the ratio of the right ventricle to the left ventricle and septum. Right ventricular hypertrophy was observed in the 3-week hypoxia group when compared with the normoxia group (25.20 ± 0.41 *vs.* 22.06 ± 0.50%, *P* < 0.005). Treatment with either low or high dose STS reduced hypoxia induced right ventricular hypertrophy when compared with the hypoxia group (23.75 ± 0.45, 22.97 ± 0.30 *vs.* 25.20 ± 0.41%, *P* < 0.05). These results indicate that STS inhibits hypoxia induced right ventricular hypertrophy and might be partly dependent on reductions of pulmonary artery pressure in consideration of the data presented in **Figure 1**. However, treatment with LY294002 did not reduce hypoxia induced right ventricular hypertrophy when compared with the hypoxia group (24.07 ± 0.58 *vs.* 25.20 ± 0.41%, *P* < 0.05) (**Figure 2**). These results suggest that a minor role is played by the PI3/Akt/mTOR signaling pathway in hypoxia-induced development of right ventricular hypertrophy when treated with LY294002. When compared with the low dose STS group, pulmonary artery pressure in the LY294002 treated group was markedly high (**Figure 1**), and might explain why LY294002 suppressed pulmonary artery pressure, but not right ventricular hypertrophy.

### Effect of Treatment With STS on Hypoxia-Induced Lung Edema

**Figure 3:**
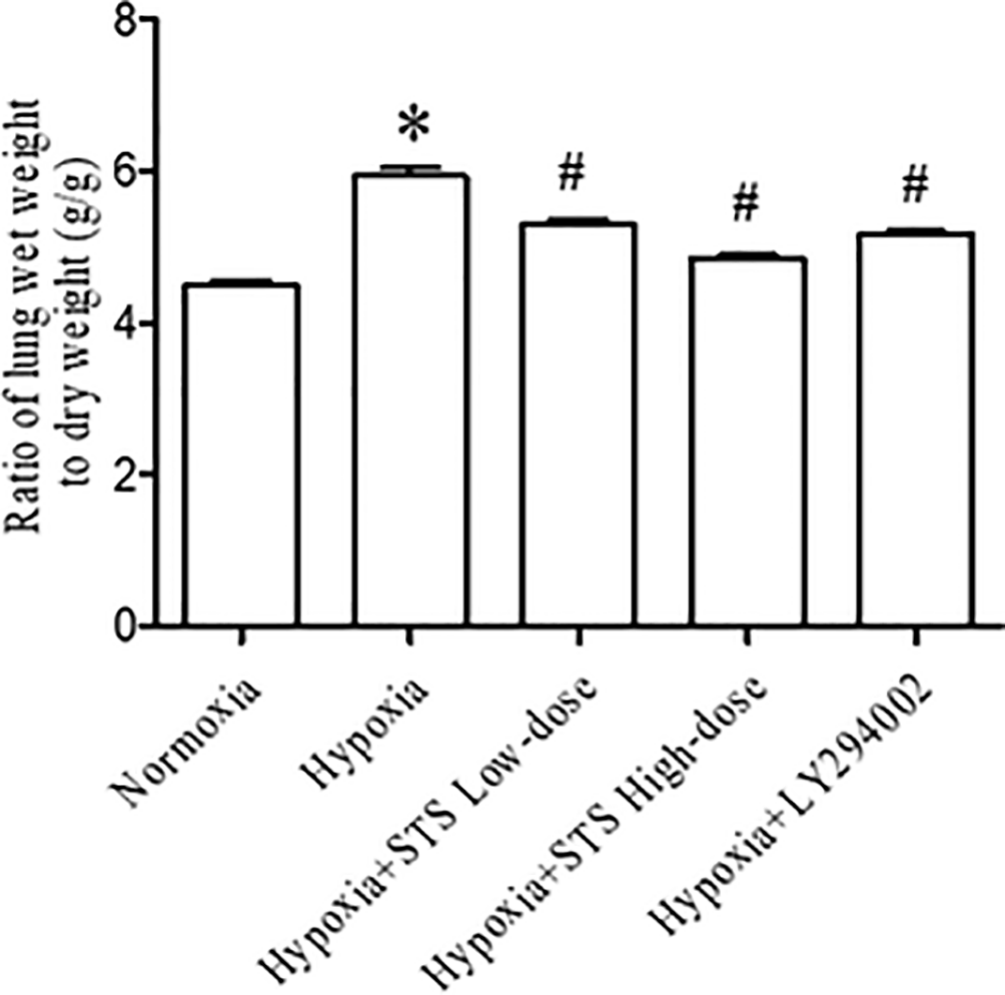
Ratio of lung wet weight to dry weight for each group. Data are presented as mean ± SEM (n = 6). * *P*< 0.05 compared with normoxia group, ^#^*P* < 0.05, compared with hypoxia group.

Results of our study demonstrated that hypoxia induced lung edema in the hypoxia group was typified by an increased ratio of lung wet weight to dry weight when compared with normoxia group (5.94 ± 0.11 *vs.* 4.50 ± 0.07, *P* < 0.001). Treatment with either the low or high dose of STS reduced hypoxia induced increases in the ratio of lung wet weight to lung dry weight when compared with the hypoxia group (5.30 ± 0.07, 4.85 ± 0.04 *vs.* 5.94 ± 0.11, *P* < 0.001). These results suggest that treatment with STS alleviated hypoxia induced lung edema. In addition, treatment with LY294002 significantly reduced hypoxia induced increases in the ratio of lung wet weight to dry weight when compared with the hypoxia group (5.13 ± 0.03 *vs.* 5.94 ± 0.11, *P* < 0.001) (**Figure 3**). This suggests that the PI3/Akt/mTOR signaling pathway may be involved in hypoxia induced lung edema.

### Effect of Treatment With STS on Hypoxia-Induced Medial Artery Thickness in Lung Tissue

**Figure 4:**
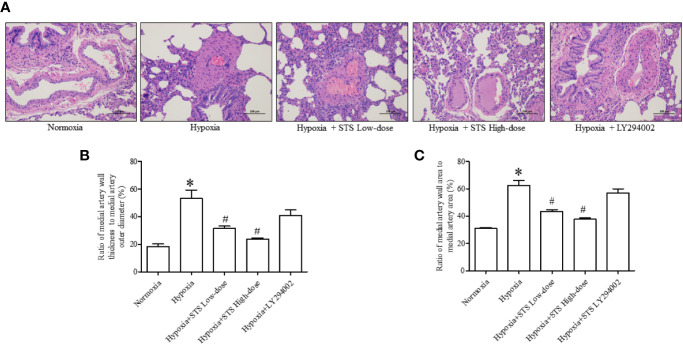
Histological analysis of lung medial artery thickness. **(A)** Representative histological images of HE staining of lung medial artery area in each group. **(B)** Ratio of medial artery wall thickness to artery outer diameter in each group. **(C)** The ratio of medial artery wall area to medial artery area of each group. Data are presented as mean ± SEM (n = 6). *P < 0.05 compared with normoxia group, ^#^*P* < 0.05, compared with hypoxia group.

Hypertrophy of the medial artery in the lung was apparent *via* histological analysis. Hypoxia increased the ratio of medial artery wall thickness to medial artery outer wall diameter (53.38 ± 6.00 *vs.* 18.30 ± 1.86, *P* < 0.001), and the ratio of medial artery wall area to medial artery area (62.52 ± 3.66 *vs.* 31.17 ± 0.60, *P* < 0.001) when compared with the normoxia group. Treatment with either low or high dose of STS markedly reduced hypoxia induced increases in the ratio of medial artery wall thickness to medial artery wall outer diameter (31.40 ± 1.75, 23.85 ± 0.63 *vs.* 53.38 ± 6.00, *P* < 0.05), and ratio of medial artery wall area to medial artery area (43.59 ± 1.21, 37.73 ± 0.94 *vs.* 62.52 ± 3.66, *P* < 0.05) when compared with the hypoxia group. In addition, treatment with LY294002 did not significantly reduce hypoxia induced increases in the ratio of medial artery wall thickness to medial artery wall outer diameter (40.62 ± 4.41 *vs.* 53.38 ± 6.00, *P* < 0.05). Treatment with LY294002 had no significant effect on hypoxia induced increases of the ratio of medial artery wall area to medial artery area (56.95 ± 3.05 *vs.* 62.52 ± 3.66, *P* < 0.05) when compared with the hypoxia group (**Figure 4**).

### Effects of STS Treatment on Lung Tissue Apoptotic Signaling Pathway

**Figure 5:**
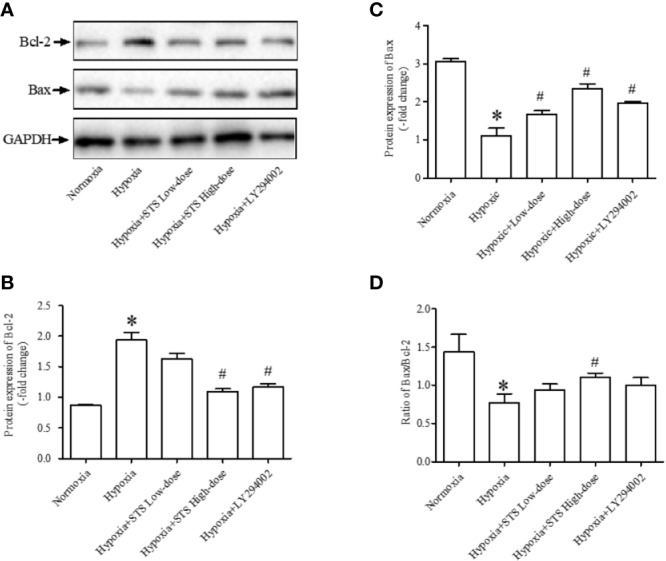
Expression of anti-apoptotic and pro-apoptotic proteins. **(A)** Representative blots of Bcl-2 and Bax proteins, GAPDH acted as the internal reference; **(B)** densitometry analysis of Bcl-2 expression in each group; **(C)** densitometry analysis of Bax expression in each group; **(D)** densitometry analysis of the ratio of Bax to Bcl-2 in each group. Each bar presents the Mean ± SEM, n = 5–8. *P < 0.05 compared with normoxia group, ^#^*P* < 0.05, compared with hypoxia group.

In the current study we observed that hypoxic conditions induced a significant increase in the expression of Bcl-2, an anti-apoptotic protein (1.94 ± 0.12 *vs.* 0.87 ± 0.01, *P* < 0.001). Exposure to hypoxic conditions decreased expression of Bax, a pro-apoptotic protein (1.11 ± 0.19 *vs.* 3.05 ± 0.09, *P* < 0.001), and decreased the ratio of Bax/Bcl-2 (0.77 ± 0.12 *vs.* 1.43 ± 0.23, *P* < 0.05) when compared with the normoxia group. Treatment with the high dose of STS partially reversed hypoxia induced increases in expression of Bcl-2 (1.10 ± 0.04 *vs.* 1.94 ± 0.12, *P* < 0.001) when compared with the hypoxia group. In addition, treatment with the high dose of STS partially reversed decreases in expression of Bax (2.35 ± 0.13 *vs.* 1.11 ± 0.19, *P* < 0.001) and ratio of Bax/Bcl-2 (1.11 ± 0.05 *vs.* 0.77 ± 0.12, *P* < 0.05) when compared with the hypoxia group. The above results suggest that the effects of hypoxia on the tissue apoptotic signaling pathway were reversed by treatment with STS. Treatment with LY294002 reversed hypoxia induced changes in Bcl-2 (1.17 < 0.05 *vs.* 1.94 ± 0.12, *P* < 0.01) and Bax (1.98 ± 0.03 *vs.* 1.11 < 0.19, *P* < 0.01) expression. However, treatment with LY294002 did not alter ratios of Bax/Bcl-2 (1.00 < 0.10 *vs.* 0.77 ± 0.12, *P <* 0.05) when compared with the hypoxia group (**Figure 5**). These results suggest that the PI3/Akt/mTOR signaling pathway plays a minor role in hypoxia induced impairment of the apoptotic signaling pathway as cells entering the apoptotic process are dependent on the balance of pro- and anti-apoptotic protein expression.

### Effect of Treatment With STS on PI3/Akt/mTOR/Autophagy Signaling Pathway

**Figure 6:**
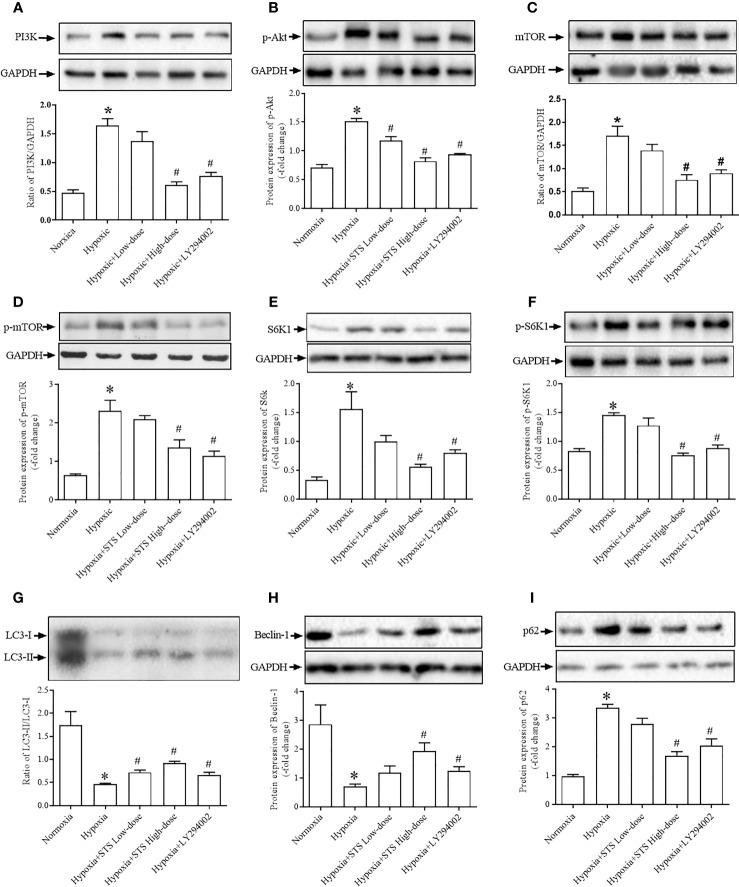
Expression of proteins of the PI3K/PAkt/mTOR/autophagy signaling pathway. **(A)** expression of PI3K, **(B)** phosphorylated Akt (p-Akt), **(C)** mTOR, **(D)** phosphorylated mTOR (p-mTOR), **(E)** S6K1, **(F)** phosphorylated-S6K1 (p-S6K1), **(G)** LC3-II/LC3-I, expressed as the ratio of LC3-II to LC3-I, **(H)** Beclin-1, **(I)** p62. Upper: representative blots of each protein, GAPDH was used as the internal reference; bottom: densitometry analysis of each protein. Each bar presents the Mean ± SEM, n = 5–8. *P < 0.05 compared with normoxia group, ^#^*P* < 0.05, compared with hypoxia group.

Results demonstrated that exposure to hypoxic conditions increased PI3 protein expression when compared with the normoxia group (1.64 ± 0.12 *vs.* 0.48 ± 0.05, *P* < 0.001). Expression of the down-stream proteins of PI3, phosphorylated-Akt (1.52 ± 0.05 *vs.* 0.71 ± 0.06, *P* < 0.01), mTOR (1.72 ± 0.20 *vs.* 0.52 ± 0.06, *P* < 0.001), and phosphorylated-mTOR (2.32 ± 0.27 vs. 0.64 ± 0.03, *P* < 0.001) were significantly higher when compared with the normoxia group. Expression of down-stream proteins of mTOR, S6K1 (1.56 ± 0.30 *vs.* 0.33 ± 0.01, *P* < 0.01) and phosphorylated-S6K1 (1.45 ± 0.04 *vs.* 0.83 ± 0.05, *P* < 0.001) were significantly greater when compared with the normoxia group. Treatment with LY294002 completely suppressed hypoxia induced increases in proteins expression of PI3 (0.77 ± 0.06 *vs.* 1.64 ± 0.12, *P* < 0.001), phosphorylated-Akt (0.94 ± 0.02 *vs.* 1.52 ± 0.05, *P* < 0.001), mTOR (0.90 ± 0.07 *vs.* 1.72 ± 0.20, *P* < 0.001), phosphorylated-mTOR (1.15 ± 0.11 vs. 2.32 ± 0.27, *P* < 0.001), phosphorylated-S6K1 (0.88 ± 0.06 *vs.* 1.45 ± 0.04, *P* < 0.001), and S6K1 (0.81 ± 0.05 *vs.* 1.56 ± 0.30, *P* < 0.05) when compared with the hypoxia group. When compared with the LY294002 treatment group, treatment with the high dose of STS resulted in similar effects on hypoxia induced increases in proteins expression. Treatment with STS resulted in decreased expression of the proteins PI3 (0.61 ± 0.05 *vs.* 1.64 ± 0.12, *P* < 0.001), phosphorylated-Akt (0.82 ± 0.06 *vs.* 1.52 ± 0.05, *P* < 0.001), mTOR (0.76 ± 0.11 *vs.* 1.72 ± 0.20, *P* < 0.01), phosphorylated-mTOR (1.36 ± 0.20 vs. 2.32 ± 0.27, *P* < 0.001), S6K1 (0.57 ± 0.04 *vs.* 1.56 ± 0.30, *P* < 0.05), and phosphorylated-S6K1 (0.75 ± 0.04 *vs.* 1.45 ± 0.04, *P* < 0.001) when compared with the hypoxia group (**Figure 6**). These results strongly suggest that treatment with STS suppressed hypoxia-induced activation of the PI3/Akt/mTOR signaling pathway.

Because mTOR negatively regulates cell autophagy as monitored *via* protein expression of LC3-II, Beclin-1 and p62, we observed evidence for autophagy in all groups. Results of our study demonstrated that exposure to hypoxia suppressed levels of lung tissue autophagy by inhibition expression of the proteins LC3-II (0.46 ± 0.02 *vs.* 1.75 ± 0.29, *P* < 0.01) and Becelin-1 (0.70 ± 0.09 *vs.* 2.87 ± 0.67, *P* < 0.05) and increased expression of the protein p62 (3.36 ± 0.11 *vs.* 0.97 ± 0.06, *P* < 0.05) when compared with the normoxia group. Our results confirm that treatment with LY294002 increased tissue autophagy by increasing protein expressions of LC3-II (0.67 ± 0.06 *vs.* 0.46 ± 0.02, *P* < 0.01) and Becelin-1 (1.25 ± 0.14 *vs.* 0.70 ± 0.09, *P* < 0.01), and decreased expression of the protein p62 (2.05 ± 0.23 *vs.* 3.36 ± 0.11, *P* < 0.05) when compared with the hypoxia group. Treatment with the high dose of STS had similar effects on hypoxia induced suppression of tissue autophagy. Treatment with STS resulted in greater expression of LC3-II (0.92 ± 0.04 *vs.* 0.46 ± 0.02, *P* < 0.01) and Becelin-1 (1.94 ± 0.28 *vs.* 0.70 ± 0.09, *P* < 0.01), and decreased expression of p62 (1.69 ± 0.14 *vs.* 3.36 ± 0.11, *P* < 0.05) when compared with the hypoxia group (**Figure 6**). These results demonstrate that treatment with STS improved hypoxia induced suppression of tissue autophagy *via* the PI3/Akt/mTOR signaling pathway.

### Effect of Treatment With STS on Hypoxia Induced Inflammatory Factors

**Figure 7:**
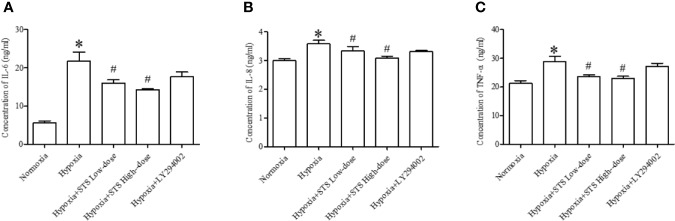
Content of inflammatory factors in each group. **(A)** IL-6, **(B)** IL-8, **(C)** TNF-α. Each bar presents the Mean ± SEM, n = 5-8. *P < 0.05 compared with the normoxia group, ^#^*P* < 0.05, compared with the hypoxia group.

In the current study we demonstrated that hypoxia increases expression of pro- inflammatory factors, IL-6 (21.79 ± 2.3 *vs.* 0.70 ± 0.09 ng/ml, *P* < 0.01), IL-8 (3.58 ± 0.13 *vs.* 2.99 ± 0.07 ng/ml, *P* < 0.01), and TNF-α (28.89 ± 1.83 *vs.* 21.19 ± 1.02 ng/ml, *P* < 0.01) when compared with the normoxia group in lung tissues of Sprague Dawley rats. Treatment with the high or low dose of STS resulted in suppression of tissue levels of IL-6 (16.02 ± 0.82, 14.23 ± 0.35 *vs.* 21.79 ± 2.3 ng/ml, *P* < 0.05), IL-8 (3.33 ± 0.16, 3.09 ± 0.06 *vs.* 3.58 ± 0.13 ng/ml, *P* < 0.01), and TNF-α (23.55 ± 0.66, 23.04 ± 0.68 *vs.* 28.89 ± 1.83 ng/ml, *P* < 0.01) when compared with the hypoxia group. However, treatment with LY294002 did not affect hypoxia induced increases in pro-inflammatory factors (**Figure 7**). These results suggest that treatment with STS attenuates the inflammatory factors in hypoxia induced pulmonary hypertension *via* a mechanism which is independent on the PI3/Akt/mTOR signaling pathway.

## Discussion

Results of the current study demonstrates that STS exerts its therapeutic effects *via* multiple mechanisms in hypoxia induced PH, as regulated by the PI3/Akt/mTOR/autophagy signaling pathway, inhibition of inflammatory factors, and lung edema.

Molecular abnormalities of PH have been widely reviewed, a key characteristic is resistance to apoptosis as observed in pulmonary arterial smooth muscle cells derived from PH models (Paulin and Michelakis, 2014). Resistance to apoptosis is likely due to suppression of tumor suppressor genes such as p53, p21, and p27, and subsequent regulation of mitochondria-dependent apoptosis. Resistance to apoptosis is highly related to tissue proliferation, especially in vessel tissues, which may contribute to arterial hypertrophy in lungs of PH patients. Results of our study demonstrated reduced apoptosis in lung tissues and increased lung medial artery hypertrophy following 3 weeks exposure to hypoxic conditions. These results were consistent with previous findings. The inhibitory effects of STS on regulation of various factors which activate apoptosis has been documented previously (Yang et al., 2008; Zhang et al., 2017). In the current study we demonstrated that treatment with STS had a pro-apoptotic effect following suppression of apoptotic levels in hypoxia induced PH, although detailed molecular mechanisms remain unclear. Treatment with STS might affect mitochondrial function, thereby up-regulating mitochondrial-dependent apoptosis (Zhou et al., 2003). In consideration of our results we propose that STS exerts dual effects on regulation of apoptosis dependent on tissue or cell conditions.

The PI3/Akt/mTOR signaling pathway plays an important role in PH, especially vascular remodeling and fibrosis (Li Y. et al., 2019; Xu et al., 2019). We observed activation of the PI3/Akt/mTOR signaling pathway in hypoxia induced PH rats, which is consistent with previous studies where anti-inflammatory effects of STS have been reported in various models (Zhu et al., 2017; Li X.X. et al., 2019; Wang Z. et al., 2019). In addition, results of our study demonstrated that treatment with STS reduced hypoxia induced increases in inflammatory factors, and concomitantly suppressed prevalence of lung edema, indicating that STS exerts its anti-PH effect *via* its anti-inflammatory effects. In the present study, we treated rats with a specific PI3/Akt/mTOR signaling pathway inhibitor, LY294002, which resulted in suppression of the development of hypoxia-induced PH, demonstrating that the PI3/Akt/mTOR signaling pathway is involved in the hypoxia induced PH. However, treatment with LY294002 did not significantly suppress hypoxia induced increases in pulmonary medial artery wall thickness, likely due to the fact that LY294002 did not reduce pulmonary pressure as strongly as STS. In addition, other signaling pathways, such as inflammatory factors might contribute to pulmonary arterial hypertrophy, and that LY294002 had lesser effects on increases in inflammatory factors when compared with STS.

The PI3/Akt/mTOR pathway negatively regulates cell autophagy and serves as a conservative protective mechanism for cellular self-digestion and basal homeostasis. The role of autophagy in hypoxia induced PH has been reviewed and has been ascribed to two aspects (Wang K. et al., 2019; Zhang et al., 2019) autophagy promotes development of hypoxia‐induced PH by activating proliferation and migration of pulmonary artery smooth cells during the early stage of PH (Zhang et al., 2014) and upregulation of mTOR partly reverses pulmonary hypertension under hypoxia (Li et al., 2015), and autophagy has a protective effect on hypoxia induced PH. Inhibition of autophagy inhibits human pulmonary artery smooth cell (HPASMC) survival (Ibe et al., 2013). In addition it has been reported that hypoxia-induced LC3B can interact with Fas by associating with caveolin-1 in lipid rafts to prevent apoptosis, thus facilitating survival of lung epithelial cells (Tanaka et al., 2012). The above-mentioned studies suggest a protective role of autophagy in PH. Although the function of autophagy in animal models of hypoxia induced PH remains unclear, the present results demonstrate that hypoxic conditions suppress lung tissue autophagy. In addition, treatment with LY294002 or STS alleviated development of PH which was accompanied by improved levels of tissue autophagy. These results suggest that STS exerted therapeutic effects on class III PH by regulating issue autophagy *via* the PI3/Akt/mTOR/autophagy signaling pathway.

Increased pro-inflammatory factors is characteristic of hypoxia induced PH (Eltzschig and Carmeliet, 2011) and is a major contributing factor in development of tissue edema. Tissue edema affects exchange of gases in lungs and can exacerbate hypoxia. Findings of this study demonstrate that increases in pro-inflammatory factors and lung tissue edema in lungs in response to hypoxia. Anti-inflammatory effects of STS have previously been reported (Zhu et al., 2017; Li X.X. et al., 2019; Wang Z. et al., 2019). Similarly, we demonstrated that treatment with STS resulted in reduced hypoxia induced increases of pro-inflammatory factors and suppression of lung edema. These results indicated that the anti-inflammatory effects exerted by STS contributed to alleviation of symptoms of class III PH. In addition, we found that the PI3/Akt/mTOR signaling pathway was not associated with hypoxia induced increases in pro-inflammatory factors. These findings are based on observations that treatment with LY294002 did not significantly suppress hypoxia induced increases in pro-inflammatory factors (**Figure 7**).

HIFs act as an important molecular mediator in hypoxia induced PH. In the current study we did not investigate if treatment with STS altered the expression of HIFs. More studies are required to investigate the effect of treatment with STS on expression of HIF. In addition, the effect of treatment with STS on transient receptor potential (Wang et al., 2013a) and Kv2.1 expression (Huang et al., 2009) in pulmonary artery smooth muscle cells were not investigated because there is an abundance of evidence existing.

## Conclusion

Results of our study demonstrates that treatment with STS alleviated hypoxia-induced pulmonary hypertension. The observed results were likely due to promotion of apoptosis, inhibition of the PI3K/AKT/mTOR signaling pathways, up-regulation of autophagy levels, and inhibition of the inflammatory responses in Sprague Dawley rats. These findings can be used to inform the clinical treatment of PH.

## Data Availability Statement

All data generated or analyzed during this study are included in this published article.

## Ethics Statement

The experiments were performed in according with the National Institutes of Health Guidelines for the Use of Laboratory Animals (NIH, publication number 85-23, revised 1996.), which were approved by and performed according to guidelines for the care and use of animals established by Soochow University.

## Author Contributions

Y-RB, YJ, L-HW, and RX performed the experiment. J-WC designed the project and provided funding. J-XQ designed the project and provided funding. G-XZ designed the project, provided funding, and wrote the manuscript.

## Funding

This work was supported by the National Natural Science Foundation of China (81470563, 81970422), the Suzhou Science and Technology Development Project (SYSD2016168, SYSD2017114), the Suzhou Young Talent Project (GSW1019061), the Gusu Talent Cultivation Project of Health (GSWS2019022), and 5th 333 Project of Jiangsu Province.

## Conflict of Interest

The authors declare that the research was conducted in the absence of any commercial or financial relationships that could be construed as a potential conflict of interest.

## Abbreviations

STS, Sodium tanshinone II sulfonate A; PAH, Pulmonary artery hypertension; HE, Hematoxylin-eosin; ELISA, Enzyme linked immunosorbent assay; PBS, Phosphate-buffered saline; RIPA, Radio immunoprecipitation assay; NO, Nitric oxide; HIFs, Hypoxia-inducible factors; PI3K, Phosphoinositide 3-kinase; mTOR, Mammalian target of rapamycin; LC3, Microtubule-associated proteins light chain 3; HE staining, Hematoxylin-eosin staining; ECL, Enhanced chemiluminescent reagent; IL, Interleukin; TNF-α, Tumor necrosis factor-α; GAPDH, Glyceraldehyde-3-phosphate dehydrogenase; SOCE, Store-operated calcium entry.
